# Fluorescent Reporters and Biosensors for Probing the Dynamic Behavior of Protein Kinases

**DOI:** 10.3390/proteomes3040369

**Published:** 2015-11-04

**Authors:** Juan A. González-Vera, May C. Morris

**Affiliations:** Cell Cycle Biosensors & Inhibitors, Department of Amino Acids, Peptides and Proteins, Institute of Biomolecules Max Mousseron (IBMM) CNRS-UMR 5247, 15 Avenue Charles Flahault, Montpellier 34093, France; E-Mail: juan-antonio.gonzalez-vera@univ-montp1.fr

**Keywords:** protein kinase, dynamics, fluorescence, biosensor, imaging

## Abstract

Probing the dynamic activities of protein kinases in real-time in living cells constitutes a major challenge that requires specific and sensitive tools tailored to meet the particular demands associated with cellular imaging. The development of genetically-encoded and synthetic fluorescent biosensors has provided means of monitoring protein kinase activities in a non-invasive fashion in their native cellular environment with high spatial and temporal resolution. Here, we review existing technologies to probe different dynamic features of protein kinases and discuss limitations where new developments are required to implement more performant tools, in particular with respect to infrared and near-infrared fluorescent probes and strategies which enable improved signal-to-noise ratio and controlled activation of probes.

## 1. Introduction—Protein Kinases are Dynamic Enzymes

Protein kinases (PKs) constitute a major class of enzymes that play major roles in a wide variety of biological signaling pathways. This class of enzymes is subdivided into receptor, membrane-associated kinases and non-receptor, cytoplasmic kinases, tyrosine kinases, serine/threonine kinases and dual specificity kinases [1]. Irrespective of their biological function, these enzymes are in constant motion in space and in time, illustrating perfectly the notion of protein dynamics.

**Figure 1:**
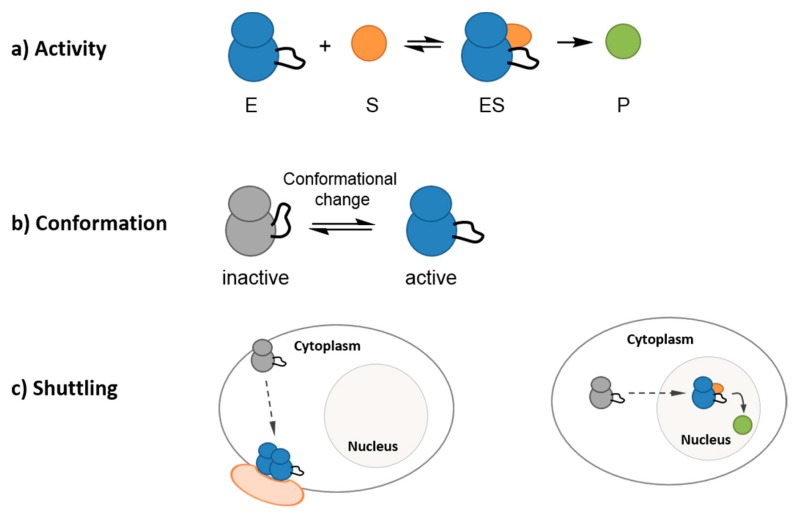
The dynamic nature of protein kinases. Protein kinases are subject to dynamic regulation of their activity, conformation and spatio-temporal localization. (**a**) Kinase activity is directly affected by its local environment, by the concentration of co-factors (nucleotides), regulatory partners and substrates, as well as by posttranslational modifications. (**b**) Protein kinase plasticity ensures conformational heterogeneity and dynamics transitions between active and inactive conformations; (**c**) These enzymes display a dynamic behavior in space and in time, undergoing dimerization within the cell membrane following specific environmental stimuli, or shuttling between different subcellular compartments.

PKs share a common structural fold, with distinct N- and C-terminal lobes, separated by a catalytic cleft, which welcomes the substrate [2,3]. However, the conformation of active kinases differs from that of inactive kinases and protein kinases undergo significant conformational transitions, which are associated with their functional and regulatory state, thereby conveying a sense of their structural plasticity [4,5,6,7,8,9]. Indeed, posttranslational modifications and interactions with regulatory partners induce changes in kinase conformation, which are intimately associated with changes in their activity, and yielding a panel of conformational states. The dynamic features of PKs are the result of several parameters in their environment, which affect their function, conformation and subcellular localization at a given point in time. Protein kinase activity is indeed subject to regulation at several levels, first by their proximity to and by the concentration of co-factors (nucleotides), regulatory partners and substrates, second by posttranslational modifications (activating and inhibitory phosphorylation, ubiquitinylation, SUMOylation, myristoylation, *etc.*), third through subcellular relocalization. Indeed, protein kinases should be viewed as macromolecules that shuttle in space and in time in a dynamic fashion in their cellular environment and between different subcellular compartments, thereby encountering different partners, evolving in environments with different pH, different molecular crowding and different viscosities, which also affect their dynamics (Figure 1). Receptor tyrosine kinases (RTKs) such as EGFR are in dynamic movement in the membrane and their heterodimerization induced upon binding of a ligand is generally associated with their activation [10]. Non-membrane kinases may shuttle between the cytoplasm and the nucleus, as exemplified by cyclin-dependent kinases CDK1/Cyclin B and CDK2/Cyclin A or E for instance, whose cyclin-mediated shuttling between cytoplasmic and nuclear compartments regulates their encounter with different substrates in a very precise and timely fashion [11,12]. Yet another variant of spatial regulation is exemplified by CDK5, anchored at the plasma membrane through association with its neurospecific partner p35, but released into the cytoplasm upon proteolytic cleavage of p35 into p25 [13].

Importantly, the vast majority of protein kinases are subject to direct or indirect alterations in their abundance, subcellular localization, activity and overall function in human pathologies, due to their own overexpression, genetic amplification, alternative splicing or mutation, or associated with alterations in regulatory partners or cofactors which influence their overall function. The development of efficient therapeutic strategies to target these dysfunctional enzymes relies on tools and technologies to undermine their alterations.

**Figure 2:**
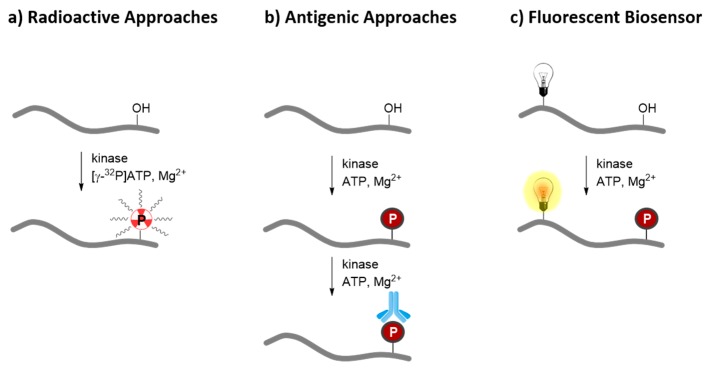
Strategies for Probing and Studying Protein Kinases *in vitro* and *in cellulo.* Several strategies have been developed to study the function/activity of protein kinases. (**a**) Radioactive endpoint assays rely on incorporation of radioactive phosphate into a substrate by the kinase; (**b**) Antigenic approaches report on phosphorylated epitopes thanks to highly specific antibodies; (**c**) Fluorescent biosensors report on phosphorylation by protein kinases through sensitive changes in fluorescence.

Given the central role of PKs in biological signaling pathways they have been the focus of numerous studies to elucidate their function and gain insight into their regulation. Moreover, since they are notoriously recognized for their deregulation in human disorders, several assays have been implemented to characterize and quantify the extent of their activity/hyperactivity (Figure 2). Traditionally, endpoint assays to assess protein kinase activity were developed by measuring incorporation of radioactive phosphate into substrates. With the development of phospho-specific antibodies to recognize specific substrates bearing phosphorylated epitopes, antigenic approaches such as Western blotting, immunofluorescence, ELISA (enzyme-linked immunosorbent assay) and immunohistochemistry, have been widely used to quantify kinase activity. However, these approaches remain discontinuous and the ability to truly monitor the dynamic features of protein kinases has only been provided by the development of fluorescent biosensors and reporters.

## 2. Fluorescent Reporters and Biosensors: Probing Kinase Dynamic Behavior in Space and in Time in a Continuous Fashion with High Temporal and Spatial Resolution

Probing the dynamic activities of enzymes, and in particular of protein kinases in a continuous fashion in space and in time constitutes a major challenge which requires specific and sensitive tools tailored to meet these particular demands, and which can further be implemented to cellular imaging. Technologies based on fluorescence offer several advantages to approach this goal. First, fluorescence lends itself to nondestructive imaging while offering a high level of sensitivity. Second, fluorescence signals may be monitored continuously over time. Moreover, fluorescence imaging enables acquisition of biomolecular behavior with high spatial and temporal resolution, thereby allowing assessing dynamic changes in activity, abundance and subcellular localization in living cells and animal models.

“Fluorescence-based” reporters or “fluorescent biosensors” are designed to recognize a target with high specificity and to report on its presence, activity or conformation with high sensitivity and in a quantitative fashion, thereby providing means to probe the biochemical function and dynamics of specific enzymes, *in vitro* and/or *in cellulo*, through sensitive changes in their fluorescent properties [14,15,16,17]. In particular, the development of fluorescent reporters and biosensors of PKs have provided a whole new avenue for studying the behavior and regulation of this class of enzymes. Such tools allow monitoring enzymatic activities in a continuous fashion *in vitro*, and in real time in living cells, following extrinsic or intracellular/natural stimuli, to study the kinetics of kinase activation, as well as response to PK inhibition in a sensitive and timely fashion. Moreover, they allow tracking the dynamic behavior of PKs in their natural/native environment in a non-invasive fashion, without perturbing the native structure or function of PK targets, and to visualize shuttling between different subcellular compartments with high spatial and temporal resolution. Additionally, fluorescent biosensors have been developed to monitor conformational changes of PKs, or to report on specific conformers [18,19,20,21]. As such, fluorescent biosensors are extremely useful in the laboratory for fundamental purposes, allowing scientists to tackle questions concerning PK function and dynamics which could not be addressed previously, both *in vitro* and in living cells in a qualitative and quantitative fashion. Beyond fundamental studies, fluorescent biosensors have also become part of the essential toolbox in drug discovery programs, as they constitute useful probes for HTS and HCS as well as for postscreen characterization of candidate drugs and assessment of their therapeutic efficacy [22]. They are equally a source of inspiration for biomedical applications, including diagnostic assays, enabling development of complementary assays or serving as alternatives to the more traditional antigenic and/or genomic approaches [23]. This is of particular relevance in the case of PK dysregulation associated with disease, since fluorescent biosensors can provide a readout of target activity, and thereby highlight alterations associated with the onset of disease, monitor changes in PK activity throughout disease progression and assess response to therapeutics, as exemplified by application of the Bcr-Abl biosensor to CML [24,25,26].

**Figure 3:**
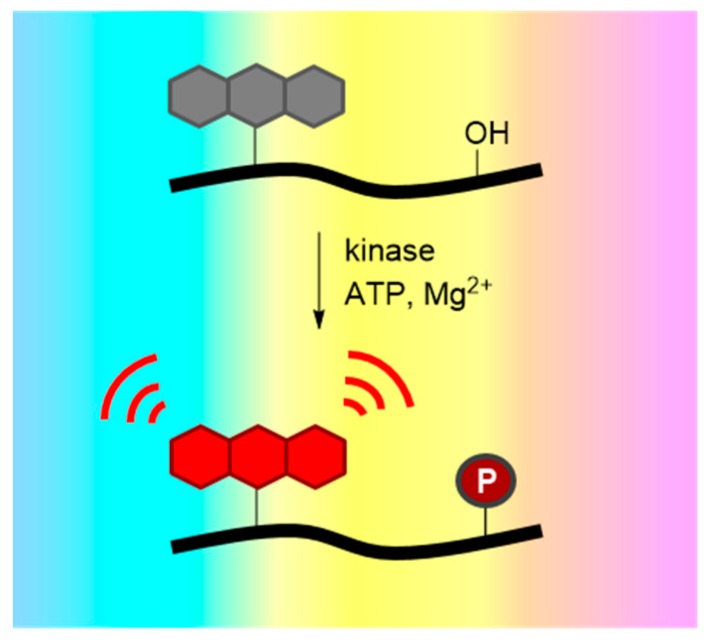
Fluorescent Kinase Biosensors. Fluorescent kinase biosensors constitute useful tools for reporting on dynamic changes in kinase activity and offer means of monitoring this process in complex biological samples, or within living cells thanks to fluorescent probes that transduce the signal into a detectable and quantifiable output. The ideal fluorescent biosensor should exhibit excellent specificity, selectivity and sensitivity when reporting on its target kinase with high signal-to-noise ratio. Ideal fluorophores for single molecule fluorescence studies have the following characteristics: (1) bright (high extinction coefficient and quantum yield of emission); (2) photostable; (3) excitable and emitting at long wavelengths, to reduce the risk of photodamage in living cells and tissues and minimize signal background; (4) highly soluble in water; (5) high labeling capability; (6) relatively small so as to introduce minimal perturbation into the host molecule; and (7) readily available in a form that can be conjugated onto the biomolecule of interest.

A wide variety of fluorescent biosensors have been developed, but all share the minimal common design of a receptor domain, which is recognized by a protein kinase (an active form, a specific conformational variant, or subcellular fraction), associated with a fluorescent probe (genetically encoded or synthetic), which will respond to this recognition event through changes in its spectral properties. Ideally, a fluorescent biosensor should recognize its target with a high level of specificity and selectivity and consequently report on its presence, activity or conformation in a quantitative fashion with a high signal-to-noise ratio. Such biosensors should be able to respond to a single protein kinase amongst the 518 different kinases in the human kinome. Alternatively, fluorescent biosensors may be designed to discriminate one conformation from another (for instance active *versus* inactive). The robustness of the response is directly associated with the physicochemical and photophysical characteristics which determine the brightness, and photostability of the probe, as well as its sensitivity to changes in the environment. The kinetics and duration of biosensor response as well as its reversibility will provide information with respect to the catalytic activity and dynamics of the target enzyme. Morphological analysis and comparison of fluorescent images with other subcellular markers will provide information on its spatial distribution (Figure 3).

Two major classes of biosensors have been developed: Genetically-encoded single-chain FRET (Förster Resonance Energy Transfer) biosensors, which rely on ectopic expression in living cells, and non-genetic fluorescent biosensors, generated through conjugation of small synthetic fluorophores (which are sensitive to changes in their environment) onto peptide/protein or polymeric scaffolds. The pros and cons of these two biosensor families have already been largely discussed elsewhere [21].

Genetically-encoded Kinase Biosensors were developed by cloning pairs of genetically-encoded autofluorescent proteins derived from Green Fluorescent Protein (GFP) into plasmid constructs together with kinase-specific substrate sequence and a phosphoamino acid-binding domain (PAABD) [16,21,27,28,29,30]. These single-chain FRET biosensors undergo reversible conformational changes upon phosphorylation by an active kinase, due to preferential binding between the PAABD and the phosphorylated form of the substrate, which in turn brings the AFPs closer, thereby enabling energy transfer between the donor and the acceptor. Genetically-encoded biosensors have been widely and successfully implemented to study PK activity in living cells. Indeed the plasmid constructs encoding these biosensors can be readily transfected into cells and changes in subcellular localization and in FRET/FLIM between donor and acceptor AFPs can be readily measured to monitor dynamic PK activities.

Equally important, several families of non-genetic kinase biosensors have been engineered through incorporation or conjugation of synthetic fluorescent dyes to peptide or protein backbones. Phosphorylation by an active kinase will affect the spectral properties of the dye either directly or indirectly. Depending on the design of the biosensor and the nature of the dye, the photophysical mechanism of response will be different, such as fluorescence enhancement, or solvatochromic shifts. These systems are more readily applicable *in vitro* but can be microinjected or introduced into living cells through facilitated delivery and have proven equally sensitive and successful to genetically-encoded kinase biosensors for monitoring protein kinase activities [17,18,19,20,21].

In this review, we will try to describe and illustrate the utility and implementation of fluorescent biosensors for monitoring different aspects of protein kinase dynamics (1) for probing kinase activity *per se* (*i.e.*, catalytic activity); (2) for monitoring their spatio-temporal subcellular dynamics; and (3) for studying conformational changes associated with PK activation. We will further discuss limitations and developments in progress to achieve yet more sensitive and performant tools.

## 3. Probing Protein Kinase Activities *in Vitro* with Peptide and Protein Biosensors

### 3.1. Peptide and Polypeptide Kinase Biosensors

Fluorescent peptide and polypeptide kinase biosensors constitute highly interesting alternatives to genetically-encoded biosensors. This class of biosensors is generated through incorporation of (a) small synthetic fluorescent label(s) into peptide substrate sequences or protein domains that bind a specific analyte or interface. This design allows for the use of a wide variety of synthetic probes with different spectroscopic properties [31], which can be incorporated at virtually any position within the peptide sequence [32], Furthermore, certain synthetic fluorophores possess photophysical characteristics that can be very attractive for the design of kinase sensors such as environmental sensitivity, chelation enhancement, *etc.* [19,20,21].

Peptide-derived biosensors offer a number of unique advantages intrinsic to their nature for the development of efficient and selective fluorescent sensors. They are relatively small, but large enough to contain a significant number of precisely located functional groups (mainly amino acid side-chains) that can encode high-affinity and specific interactions with the target protein. Moreover, during synthesis they can be modified to incorporate non-degradable biomimetic substituents, quenching or photoactivatable groups. Different strategies have been employed to develop peptide-based sensors of PK activity, which we will discuss here according to the mechanism that characterizes their response to phosphorylation: Environment-sensitive biosensors, quenching-based biosensors, and metal-ion mediated biosensors. These biosensors and their properties are summarized in Table 1. Further information may be found in the original papers or in comprehensive reviews on this class of sensors [15,19,21,33,34,35].

**Table 1:** Properties of peptide-based fluorescent sensors of protein kinase activity.

Sensing Mechanism	Fluorophore	Protein Kinase	Assay ^a^	Ref.
**Environment-Sensitive Biosensors**				
Probe proximal to phosphorylation site	NBD	PKC	*In vitro* (RE/CE); *in cellulo* to monitor the spatiotemporal dynamics of the PKC pathway	[36]
Phosphorylation-driven protein-protein interaction, based on an SH2 domain	NBD or Dapoxyl	Src	*In vitro* (RE)	[37]
CDKACT	Cy3	CDK/Cyclin activity	*In vitro* (RE/CE); *in cellulo* probing dynamics and quantification of kinase activity	[38]
Merobody: fibronectin monobody conjugated to a probe	mero53	Src	*In vitro* (CE); *in cellulo* quantification of Src activity at the edge of living cells, in correlation with protrusion and retraction activities	[39]
**Quenching-Based Biosensors**				
Self-reporting biosensor: tyrosine quencher	Pyrene	Src	*In vitro* (RE)	[40]
	Cascade Yellow, Cascade Blue or Oregon Green	Src	*In vitro* (RE); *in cellulo* probing Src activation in response to stimulation	[41]
	Cascade Yellow or Oxazine	Abl, Lyn	*In vitro* (RE); *in cellulo* simultaneous visualization of Abl and Lyn kinases in chronic myelogenous leukemia drug-resistant cell lines	[42]
Deep quench: probe/quencher/14-3-3 phosphoserine binding domain	Pyrene/Rose Bengal	PKA	*In vitro* (RE)	[43]
	Coumarin/Acid Green	PKA	*In vitro* (RE)	[44]
Quenching: probe/quencher	5Fam, TAMRA, Atto620, Atto633 or Red681/Acid Blue or Evans Blue	PKA	*In vitro* (RE/CE); *in cellulo* to monitor endogenous cAMP-dependent protein kinase activity in erythrocytes	[45]
**Metal-Ion Mediated Biosensors**				
Ca^2+^-dependent	Fluorescein	PKCα	*In vitro* (RE)	[46]
Mg^2+^-dependent, BTF	Sox	PKA, PKC, Abl	*In vitro* (RE)	[47]
Mg^2+^-dependent, BTF, cell lysates	Sox	Akt, PKA, MK2	*In vitro* (CE)	[48]
Mg^2+^-dependent, BTF, multiplexed assay in cell lysates	Sox	PKC, PKA, Akt1, MK2, CDK2, Pim2	*In vitro* (CE)	[49]
Mg^2+^-dependent, RDF	Sox	PKC, Pim2, Akt1, MK2, PKA, Abl, Src, IRK	*In vitro* (RE)	[50]
Mg^2+^-dependent, RDF, protein-based docking domain (Sox-PNT)	Sox	ERK1/2	*In vitro* (CE)	[51]
Mg^2+^-dependent, RDF, protein-based docking domain	Sox	p38α	*In vitro* (CE)	[52]
	Sox	ERK1/2, p38α/β and JNK1/2/3	*In vitro* (CE)	[53]
Lanthanide-based biosensor	Tb^3+^/Eu^3+^ (Carbostyril 123)	Src, Abl	*In vitro* (RE)	[54]
**Photoactivatable Biosensors**				
Probe proximal to phosphorylation site, caged serine	NBD	PKC	*In cellulo* to monitor PKC activity in HeLa cells following microinjection and selective photoactivation	[55]
Probe proximal to phosphorylation site, caged serine	NBD	PKCβ	*In cellulo* to monitor PKCβ activity throughout mitosis in PtK2 cells	[56]
Self-reporting biosensor, caged tyrosine	Cascade Yellow	Src	*In cellulo* to follow the timing of kinase activity following microinjection and photoactivation in A549 cells	[41]
Quenching: probe/quencher, caged serine	Atto633/Evans Blue	PKA	*In cellulo* to monitor endogenous cAMP-dependent protein kinase activity in erythrocytes following microinjection and photoactivation	[45]

^a^ RE = recombinant enzyme, CE = cell extracts.

### 3.2. Environment-Sensitive Kinase Biosensors

Environment-sensitive (also named “solvatochromic”) fluorophores exhibit alterations in their photophysical properties in response to changes in the polarity of the surrounding environment [32,57]. Most of these dyes are donor-acceptor systems with large excited-state dipole moments, being poorly fluorescent in aqueous solution and polar solvents, but becoming highly fluorescent in non-polar solvents, or upon docking into a hydrophobic protein pocket or membrane. The behavior of these “smart molecules” is well suited for biosensing applications, in particular for detection of molecular interactions. The features of several solvatochromic fluorophores and their application to monitor protein/protein interactions have been described in detail [32]. Even though most of these probes possess excellent photophysical properties for *in vitro* applications, their absorption and emission wavelengths tend to be incompatible with *in vivo* applications. Therefore, current efforts in this field are directed to the development of brighter solvatochromic fluorophores, which feature red-shifted absorption and high solvent sensitivity for *in vivo* sensing applications.

**Figure 4:**
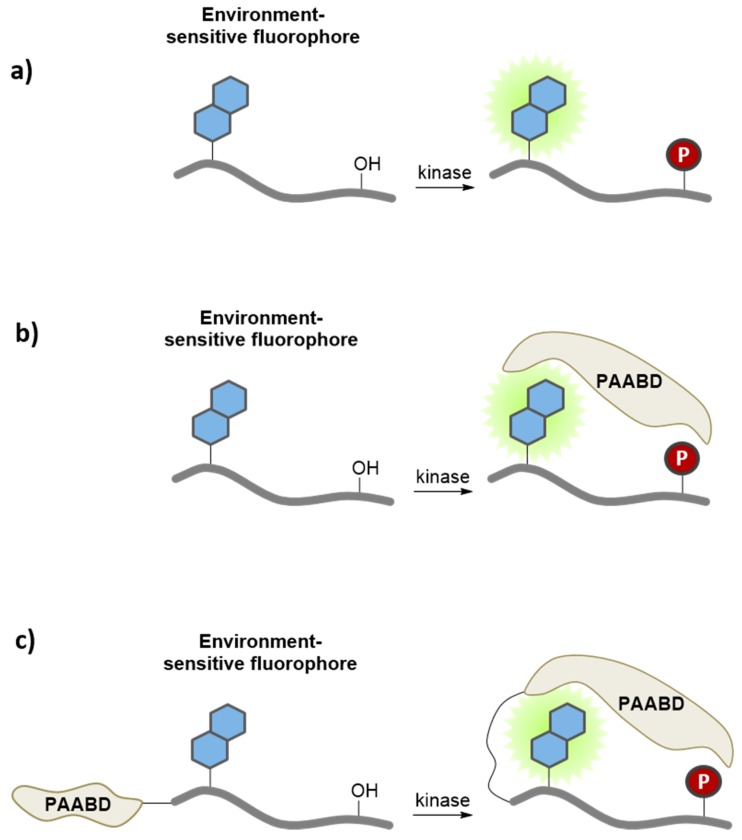
Environment-sensitive kinase biosensors. Environment-sensitive kinase biosensors are composed of a peptide sequence derived from a substrate and a fluorophore that is conjugated immediately adjacent or proximal to (*i.e.*, 2–5 residues away) the phosphorylation site. Phosphorylation modifies the local environment of the solvatochromic fluorophore, promoting (**a**) direct changes in its fluorescence emission; (**b**) indirect changes in fluorescence, following intermolecular interaction with a phospho amino acid binding domain (PAABD); or (**c**) intramolecular interaction when the PAABD is fused/conjugated to the substrate domain.

Environment-sensitive kinase biosensors are composed of a kinase recognition domain, most often a substrate, and a fluorophore that is usually conjugated immediately adjacent or proximal (*i.e.*, 2–5 residues away) to the phosphorylation site. Phosphorylation modifies the local environment of the solvatochromic dye, thereby promoting changes in its fluorescence emission (Figure 4a). For instance, a solvatochromic nitrobenzofuran NBD-peptide biosensor to probe PKC activity was generated by conjugating the NBD dye at the N-terminal serine residue of the peptide [36]. This PKC biosensor displayed a robust increase in fluorescence intensity upon phosphorylation by the enzyme *in vitro*. Alternatively, since solvatochromic fluorophores are very useful reporters of protein/protein interactions, they may be incorporated into the substrate sequence of the biosensor, so as to respond to inter- or intramolecular phosphoserine/threonine or tyrosine- binding domains, such as 14-3-3, SH2 or WW domains, following phosphorylation of the substrate by the kinase of interest (Figure 4b) [58,59,60,61,62,63]. This strategy was employed to generate a very sensitive biosensor for Src kinase activity, by combining a fluorescent peptide labeled with dapoxyl, NBD or Cascade yellow with the SH2 domain, leading to a significant increase in fluorescence emission intensity [37]. The CDKACT biosensor developed to probe cyclin-dependent kinase activity constitute yet another example [38]. This protein biosensor comprises a sequence derived from histone H1, a substrate recognized and phosphorylated by most CDK/Cyclins, which is coupled to a cyanine dye, and a phosphopeptide recognition domain derived from Plk kinase (Figure 4c). The CDKACT biosensor allows monitoring CDK/Cyclin activity in a continuous fashion, allowing comparing the catalytic activities of different recombinant CDKs and the overall endogenous activities of extracts from different cell lines.

### 3.3. Quenching Based Kinase Biosensors

**Figure 5:**
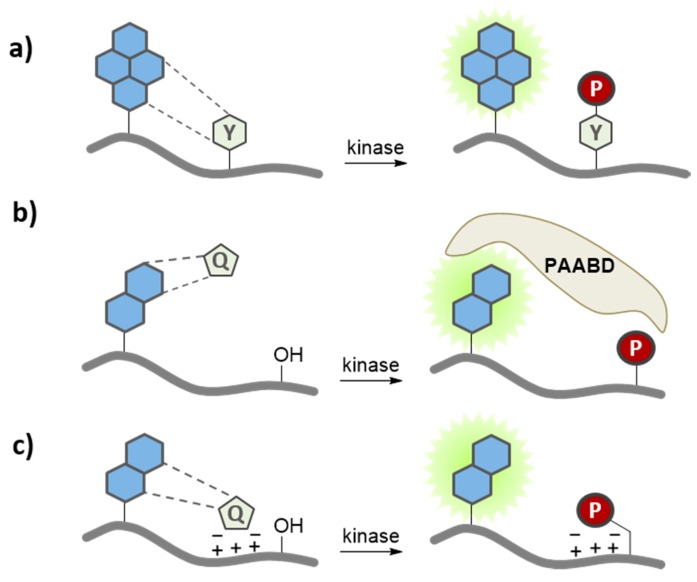
Quenching-based kinase biosensors. These biosensors exhibit differential fluorescence following phosphorylation due to, (**a**) disruption of intramolecular quenching of the fluorescent probe conjugated to a positively charged (+) substrate domain with a tyrosine residue (Y); (**b**) displacement of a soluble quencher (Q) by a PAABD that binds the phosphorylated substrate; or (**c**) displacement of a negatively charged quencher (−) due to charge repulsion upon phosphorylation (−).

Tryptophan and tyrosine are known to quench the overall fluorescence of a variety of organic fluorophores through intramolecular π-π stacking interactions, thereby reducing their fluorescence quantum yield [64,65]. This approach was explored to generate a pyrene-based biosensor to probe the protein tyrosine kinase Src. Phosphorylation of the tyrosine residue disrupts the dye-tyrosine stacking interaction resulting in a significant enhancement in fluorescence emission (4.7-fold) (Figure 5a) [40]. An alternative quenching strategy was further developed for Ser/Thr kinases, in which the fluorescence of a labeled kinase substrate was silenced by a non-covalent quencher molecule in solution (Figure 5b). Upon phosphorylation by PKA, the corresponding phosphopeptide binds to a phosphorecognition domain (such as 14-3-3π), which competes with and displaces the non-covalent quencher, therefore disrupting quenching and resulting in significant fluorescence enhancement (64-fold for a Pyrene/Rose Bengal-based biosensor; 152-fold for Coumarin-Acid Green 27-based biosensor) [43,44]. More recently, a much simpler and versatile biosensor to probe PKA activity in the NIR region has been reported (Figure 5c). In this biosensor, a positively charged fluorescent peptide, including the kinase recognition domain, is quenched upon exposure to a negatively charged quencher dye. After phosphorylation, the newly incorporated phosphate group acts as a molecular trigger that prompts release of the quencher, consequently leading to an increase in fluorescence (up to 104-fold) [45]*.*

### 3.4. Metal-Ion Mediated Kinase Biosensors

**Figure 6:**
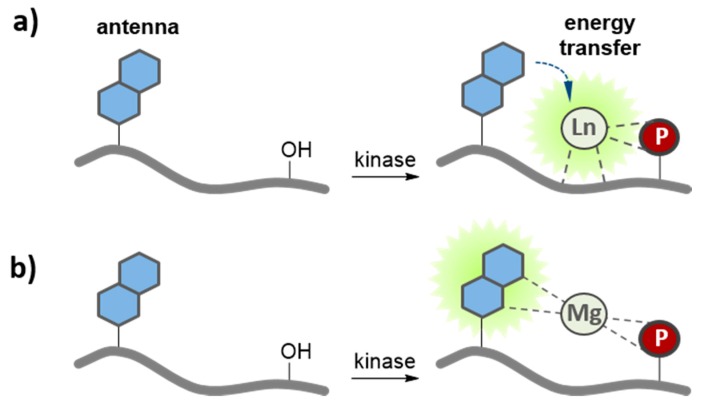
Metal-ion mediated kinase biosensors. In the unphosphorylated state, these biosensors exhibit poor metal ion affinity and are essentially non-fluorescent. In contrast, phosphorylation provides an additional ligand, therefore increasing the avidity for binding of metal ions which promotes changes in fluorescence/luminescence, (**a**) chelation and excitation of lanthanide by a sensitizer; and (**b**) magnesium chelation-enhanced fluorescence (CHEF).

Several strategies that rely on metal ion-mediated luminescence or fluorescence to sense phosphorylation have been described. In the unphosphorylated state, these biosensors exhibit poor metal ion affinity and are essentially non-fluorescent. However, incorporation of a phosphate group provides two essential ligands, resulting in a significant increase in the avidity for binding to hard metal ions, such as magnesium or lanthanide ions, and a subsequent positive signal. Several peptide biosensors have been devised to bind lanthanide ions such as Tb^3+^ or Eu^3+^ [54,66,67]. In these cases, the mandatory nearby antenna (Trp or carbostyril) absorbs light and sensitizes the corresponding lanthanide, which produces long-lived luminescence to sense kinase activity (Figure 6a). Upon phosphorylation, these biosensors display up to 10-fold increase in luminescence. Unfortunately this strategy is mainly restricted to *in vitro* conditions due to the requirement of the free cell-toxic lanthanide ions.

On the other hand, chelation-enhanced fluorescence (CHEF) sensing methods utilize biologically available metal ions, such as Ca^2+^ and Mg^2+^, to probe kinase activity. The Lawrence group described a kinase biosensor, exploiting elevated Ca^2+^ levels required for proper PKC_α_ activation [46]. This modular sensing system includes a kinase recognition domain and a previously described fluorescein-based Ca^2+^ indicator, directly attached to the N-terminal phosphorylation site. The unphosphorylated probe has low affinity for Ca^2+^, but phosphorylation increases the binding affinity of the sensor promoting a 3.6-fold increase in fluorescence. However, this approach has not been adapted for use with Mg^2+^, required for activation of the majority of kinases. As an alternative Imperiali and collaborators developed powerful sensors containing the phosphorylation-sensitive fluorescent amino acid Sox [68] to monitor kinase activity in the presence of physiological levels of Mg^2+^ (Figure 6b). The Sox-based fluorescent peptides have a modular design that incorporate the Sox amino acid, at position ±2 relative to the Ser/Thr/Tyr phosphorylation site, and a phosphorylatable kinase recognition domain, including a β-turn peptide sequence that preorganizes a binding site for the interaction between Mg^2+^ and the Sox fluorophore. Phosphorylation at a proximal residue dramatically increases the affinity of Sox for Mg^2+^, leading to an increase in fluorescence (up to 5-fold), due to chelation of this ion between the newly incorporated phosphoryl group and the Sox fluorophore (CHEF). This sensing method, termed β-turn focused (BTF) design was successfully applied for the continuous fluorescence-based monitoring of tyrosine and serine/threonine activities, both with recombinant enzyme and cell extracts [47,48,49]. To further increase the selectivity of recognition, this strategy was extended through the development of a second-generation cysteine derivative of the Sox fluorophore, named C-Sox. The increased flexibility of C-Sox allows for incorporation of both N- and C-terminal kinase recognition elements into second-generation probes, leading to improved selectivity and kinetic properties. This recognition-domain focused (RDF) design, allowed the construction of chemosensors for a variety of well-characterized Ser/Thr and Tyr kinases, including PKC, Pim2, Akt1, MK2, PKA, IRK, Src and Abl, with robust enhancements (up to 10-fold) and high sensitivity under a variety of conditions [50]. Moreover, high-throughput approaches were developed to generate Sox-based chemosensors with improved photophysical properties [69] and the highest possible selectivity [70], using combinatorial libraries. More recently, the RDF strategy was extended to improve the specificity of Sox biosensors through ligation of a protein docking domain. A Sox-based ERK1/2 sensor (Sox-PNT) composed of the PNT domain from the Ets1 transcription factor and the Sox-ERK1/2 peptide substrate sequence was developed and successfully applied to probe ERK1/2 kinase activity in cell lysates, displaying high selectivity against other kinases from the JNK, p38, and CDK families [51]. Similarly, Sox-based chemosensors for three MAP kinase subfamilies (ERK1/2, p38α/β, and JNK1/2/3) were reported to selectively probe kinase activity in cell extracts thanks to peptide docking domains, which confer high affinity/selectivity for the target enzyme [52,53].

### 3.5. Application of Peptide and Protein Biosensors of PKs in Living Cells

One of the major challenges for application of peptide biosensors in living cells consists in introducing them into living cells. Indeed uptake of peptides and probes is not trivial, since biological membranes constitute efficient barriers for biomolecules. Moreover, once delivered through the cell membrane these biomolecules must be able to reach and recognize their intracellular target. This being said, a wide variety of strategies have been devised and optimized to deliver peptides, proteins and probes into cells over the last decade. Hence the lack of permeability of the cell membrane should no longer be considered a major challenge for this class of biosensors.

Besides microinjection, electroporation or cell-loading approaches which apply mechanical stress on the cell membrane [71], transduction of peptide and protein biosensors into living cells can be achieved thanks to peptide-based carriers such as protein-transduction domains or cell-penetrating peptides, which are either covalently fused (e.g., TAT), conjugated or complexed into non-covalent nanoparticles [72,73,74,75]. In addition lipid-based formulations, cationic and polymeric carriers, as well as silica-based nanoparticles or carbon nanotubes offer plenty of platforms to ensure delivery of peptide and protein biosensors into cells [76,77,78].

Several peptide-based biosensors have been successfully applied to monitor protein kinase activities in living cells. For instance, the solvatochromic NBD-based biosensor for PKC (described above) was successfully microinjected into living cells to visualize and monitor the spatiotemporal dynamics of PKC activity.

Gulyani *et al.* generated a solvatochromic Src biosensor based on a fibronectin monobody scaffold, which was employed to quantify the dynamics of Src activity at the edge of living cells, in correlation with protrusion and retraction activities [39]. This merobody biosensor was engineered by an HTS approach, by derivatizing a monobody that specifically recognizes the active conformation of Src kinases with an environmentally-sensitive merocyanine dye, the fluorescence of which is enhanced upon target binding, yet without interfering with target binding or phosphorylation. Furthermore, variants of the above-mentioned “self-reporting” biosensor labeled with Cascade Yellow, Cascade Blue, Oregon Green or Oxazine were successfully applied to probe Src, Abl or Lyn *in cellulo* through microinjection [41,42]. Likewise, the quenching-based NIR biosensor for PKA (described above) was effectively employed *in cellulo* following microinjection [45].

More recently, the CDKACT protein-biosensor was introduced into living cells through complexation with cell-penetrating peptides that form nanoparticles with their cargo and ensure its efficient and non-covalent intracellular delivery [38,74]. These formulations enabled application of CDKACT to monitor oscillations in CDK/Cyclin activity throughout the cell cycle by time-lapse imaging and ratiometric quantification of Cy5 and RFP fluorescence, the former conjugated to the substrate sequence and responding to kinase activity, the latter serving as an intramolecular control.

## 4. Probing Protein Kinase Activities in Living Cells with Genetically-Encoded FRET Biosensors 

Since the initial cloning of GFP, a wide variety of autofluorescent protein derivatives have been developed and optimized to improve brightness, quantum yield and pH-insensitivity, so as to yield donor/acceptor couples that may transfer fluorescence resonance energy as efficiently as possible within single-chain FRET biosensors [28,79,80,81,82,83,84,85,86,87]. Although several different AFP pairs have been used for FRET biosensors, the most widely used FRET couples employed for PK biosensors are undoubtedly YFP/CFP and EGFP/mRFP or Cherry. With the development of brighter and more performant AFPs, such as Venus, mTurquoise, Clover and mRuby, new generations of FRET biosensors have been engineered and applied to image enzymatic activities.

**Figure 7:**
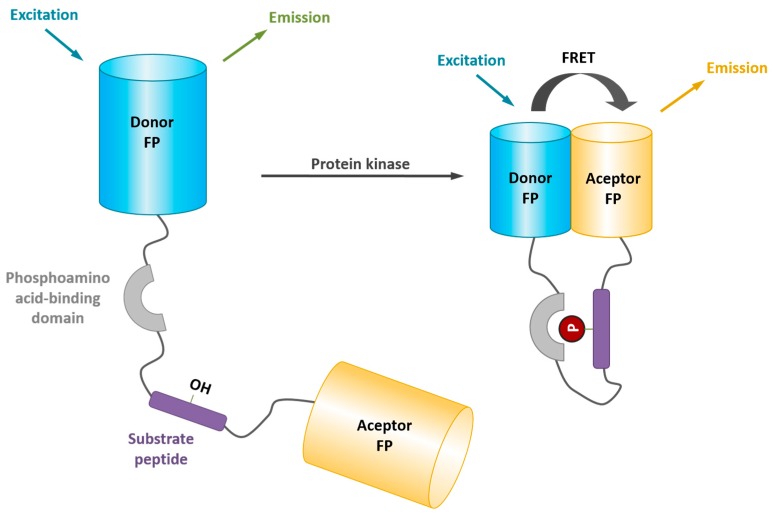
Genetically-encoded kinase biosensors. Genetically-encoded protein kinase biosensors are single-chain FRET biosensors, also known as Kinase Activity Reporters (KARs), that express a pair of genetically-encoded autofluorescent proteins (AFPs) flanking a substrate sequence and a phosphoamino acid-binding domain (PAABD) joined by a linker. In the presence of active kinase, which phosphorylates the substrate sequence of the biosensor, the PAABD is prompted to bind the phosphorylated substrate, thereby bringing the AFPs in close proximity. This intramolecular conformational change favors fluorescence resonance energy transfer between the donor and the acceptor, which results in an increase in fluorescence intensity and lifetime of the acceptor, and a concomitant decrease in the fluorescence intensity and lifetime of the donor.

Genetically-encoded protein kinase biosensors are single-chain FRET biosensors, also known as Kinase Activity Reporters (KARs), that express a pair of genetically-encoded autofluorescent proteins (AFPs) flanking a substrate sequence and a phosphoamino acid-binding domain (PAABD) joined by a linker. In the presence of active kinase which phosphorylates the substrate sequence of the biosensor, the PAABD is prompted to bind the phosphorylated substrate, thereby bringing the AFPs in close proximity. This intramolecular conformational change favors fluorescence resonance energy transfer between the donor and the acceptor, which results in an increase in fluorescence intensity and lifetime of the acceptor, and a concomitant decrease in the fluorescence intensity and lifetime of the donor (Figure 7). Several successful examples of KARs have been reported, allowing monitoring oscillations in kinase activity throughout the cell division cycle, or in specific signaling pathways with high spatial and temporal resolution. These biosensors and their properties are summarized in Table 2 and reviewed in detail in Ref. [21].

The first genetically-encoded protein kinase biosensors were developed to probe tyrosine kinases based on fusions of CFP/YFP with the SH2 phosphotyrosine-binding domain and substrate sequences recognized by Src, Abl or EGFR [88,89]. Following these first successful developments, optimized derivatives were developed, such as the Picchu sensor to probe cAbl, and the Pickles sensor to probe Bcr-Abl activity, which exploits ECFP and Venus (variant of YFP) [24,89]. Pickles has more recently been applied to assess Bcr-Abl activity in cells from patients, response to drugs and development of resistance to drugs [24,25,26].

**Table 2:** Properties of genetically-encoded FRET (Förster Resonance Energy Transfer) biosensors of protein kinase activity.

Protein Kinase	Biosensor Name	AFP FRET Pairs	Cellular Process	Ref.
Abl/EGFR	CrkII-based reporter	CFP/YFP	Rapid, dynamic and transient phosphorylation by CrkII upon epidermal growth factor stimulation.	[88]
c-Abl	Picchu	CFP/YFP	Specific phosphorylation by c-Abl.	[89]
Bcr-Abl	Pickles	ECFP/Venus	Clinical diagnosis of Bcr-Abl activity in CML patient cells: monitoring disease status, response to therapy, and the onset of drug-resistance within a heterogeneous population. Comparative assessment of inhibitor efficacy: evaluation of second generation inhibitors or novel compounds to treat drug-resistant mutants.	[24]
Aurora B	Aurora B sensor	CFP/YFP	Dynamics of Aurora B activity during anaphase.	[90]
AKT	AktAR	Cerulean/cpVenus	PKB/Akt signaling and dynamics in living cells. Spatiotemporal analysis of differential Akt regulation in plasma membrane microdomains.	[91]
AKT	AktUS	CFP/YFP	PKB/Akt dynamics in living cells, in the Golgi and mitochondria	[92]
AKT	BKAR	ECFP/Citrine	Spatio-temporal dynamics of PKB/Akt activity in real time in living cells, in the nucleus, cytosol, and plasma membrane.	[93]
AMPK	AMPKAR	ECFP/cpVenus	Probing AMPK activity upon cellular stress.	[94]
ATM	ATOMIC	CFP/YFP	Monitoring ATM kinase activity in living cells and in response to double strand breaks.	[95]
CAMKII	Camui	CFP/YFP	Activation of calcium/calmodulin-dependent protein kinase II in living neurons and in cardiomyocytes	[96]
CDK1/CyclinB activity	CDK1 sensor	mCerulean/YPet	Progressive activation of CyclinB1-Cdk1 at the G2/M transition in living cells, just before nuclear envelope breakdown, contributing to initiate prophase.	[97]
ERK	EKAR	EGFP/mRFP1	Spatiotemporal signaling dynamics of ERK kinase in HEK293 cells after epidermal growth factor stimulation, in neurons from intact brain tissue by fluorescence lifetime imaging, in the dendrites and nucleus of hippocampal pyramidal neurons in brain slices after theta-burst stimuli or trains of back-propagating action potentials.	[98]
ERK1	Erkus	CFP/YFP	Spatiotemporal dynamics of cytosolic and nuclear activity of ERK in living cells	[99]
IR	Phocus	CFP/YFP	Phosphorylation by the insulin receptor in living cells.	[100]
JNK Kinase	JNKAR	EGFP/citrine	Spatiotemporal dynamics of JNK activity-signaling properties and behavior of the JNK cascade in living cells.	[101]
FAK Kinase	FAK sensor	ECFP/YPet	Focal adhesion kinase activity and activation at membrane microdomains.	[102]
Histone Phosphorylation		CFP/YFP	Histone phosphorylation in living cells.	[103]
MLCK	MLCK-FIP (Ca^2+^/calmodulin)	CFP/YFP	Transient and regional myosin light chain kinase activation in lamella and cleavage furrows. Spatial and temporal pattern of MLCK activation, revealing enrichment at the spindle equator during late metaphase and maximal activation just before cleavage furrow constriction.	[104]
PKA	ART	BGFP/RGFP	cAMP-induced dynamics of PKA activation in COS-7 transfected cells.	[105]
PKA	AKAR1	ECFP/YFP	PKA activity following substrate tethering.	[106]
PKA	AKAR2	ECFP/Citrine	Insulin disrupts β-adrenergic signaling to protein kinase A in adipocytes.	[107]
PKA	AKAR	EGFP/cpVenus	Subcellular dynamics of PKA activity.	[108]
PKA	AKAR3	CFP/YFP	Detection of dynamic PKA activity in the sarcoplasmic reticulum of cardiomyocytes.	[109]
PKC	CKAR	ECFP/Citrine	Oscillatory activity of PKC at the plasma membrane in response to histamine, associated with calcium oscillation.	[110]
PKC-delta	deltaCKAR	CFP/YFP	Monitoring PKCdelta activity.	[111]
PKC	KPC-1 (pleckstrin based)	GFP/EYFP	PKC activation through phorbol ester stimulation or upon activation of physiologically relevant pathways	[112]
PKA and PKC	KPAC-1 (pleckstrin based)		Monitoring PKA and PKC activities independently in living cells.	[113]
PKD	DKAR	CFP/YFP	Monitoring protein kinase D dynamics and its dependence on calcium through positive feedback regulation of diacylglycerol production.	[114]
Plk1	Plk sensor	CFP/YFP	Mitotic Plk1 kinase activity in human cells in a physiological context and upon checkpoint recovery.	[115]
SAP3K	SAP3K activity reporter	Venus/SECFP	Stimulus-specific distinctions in spatial and temporal dynamics of SAP3K activity towards MKK6 SAP2K in living cells: response to epidermal growth factor and osmostress at the plasma membrane, anisomycin and UV in the cytoplasm, etoposide in the nucleus.	[116]
Src	Src sensor	CFP/YFP	Dynamics of Src activation following mechanical stimuli.	[117]
c-Src	Srcus	CFP/YFP	Src activation by steroids in the cytosol and at the plasma membrane. Epidermal growth factor directs sex-specific steroid signaling through Src activation.	[118]
Syk	Syk sensor	ECFP/Ypet	Imaging and quantifying real-time activation of Syk upon immunoreceptor activation and following stimulation by the platelet-derived growth factor.	[119]
ZAP-70	ROZA	CFP/YFP	Dynamics of the ZAP-70 tyrosine kinase activity in T-cell lines and primary human lymphocytes with subcellular resolution during the formation of an immunological synapse.	[120]
MARK	MARK sensor	ECFP/Citrine	Evaluation of microtubule affinity regulating kinase activity in living neurons.	[121]
RSK	Eevee-RSK	ECFP/Ypet	Probing RSK activity and quantitative evaluation of kinase inhibitors in living cells.	[122]
S6K	Eevee-S6K	Turquosie-GL/Ypet	Probing S6K activity and quantitative evaluation of kinase inhibitors in living cells.	[122]

A biosensor of the non-receptor tyrosine kinase Src was developed based on an ECFP/YFP couple, an SH2 domain and a substrate peptide derived from the c-Src substrate p130cas, and applied to image the dynamics of Src activation following mechanical stimuli [117]. The Srcus of this biosensor including a nuclear export signal served to study Src activation by steroids mediated by EGFR [118].

A FRET-based biosensor of the spleen tyrosine kinase Syk was engineered by incorporating an ECFP/YPet FRET couple, an SH2 domain and a substrate sequence derived from VAV2, and applied to image real-time activation of Syk in living cells upon immunoreceptor activation and following stimulation by platelet derived growth factor [119].

A single-chain FRET biosensor of ZAP-70 was developed thanks to a CFP/YFP FRET pair, the SH2 domain of Grb2, and a substrate sequence derived from LAT. ROZA (Reporter Of ZAP-70 Activity) was applied to image tyrosine kinase activity of ZAP-70 in T-cell lines and primary human lymphocytes [120].

Several genetically-encoded FRET biosensors of PKA have been developed and this kinase has been widely studied in living cells thanks to these imaging tools, in particular the AKAR biosensors developed by Zhang *et al.* which incorporated first a 14-3-3, then an FHA domain as a PAABD to recognize the consensus PKA phosphorylation site [105,106,107,108,123].

Several genetically-encoded FRET biosensors of PKC have been developed [110,111,112,113]. CKAR incorporates a consensus phosphorylation sequence and the FHA2 domain of Rad53 sandwiched between a mCFP/mYFP pair [110], and more recent variants can discriminate between different PKC isoforms [111]. The KCP-1 biosensor comprises a PH domain (Pleckstrin homology) and a DEP domain (Dishevelled, Egl-10, Pleckstrin) separated by an intervening PKC substrate sequence, phosphorylation of which induces an intramolecular conformational change between the PH and DEP domains [112]. The KCAP-1 derivative includes an additional PKA substrate sequence, which makes it capable of measuring both PKC and PKA activities independently, responding positively to PKC phosphorylation (increase in FRET) and conversely to PKA [113].

Several fluorescence-based reporters of PKB/Akt have been engineered and applied to monitor kinase activity in living cells [91,92,93]. Aktus reporters are constructed similar to AKAR biosensors, with a PKB substrate sequence derived from Bad and a PAABD domain derived from 14-3-3n, while the BKAR sensor resembles CKAR. Yet another PKB reporter, AktAR, was developed through incorporation of an FHA1 domain and a FOXO1 substrate into a construct expressing the Cerulean/cpVenus couple [91].

Several KARs of ERK/MAPK kinases have also been developed. The first of these, Erkus, is a single-chain biosensor that comprises the CFP/YFP FRET pair, an FHA2 domain from Rad53p, and a substrate domain derived from EGFR T669 peptide, as well as a short docking motif, derived from p90 ribosomal S6 kinase [99,124]. Another biosensor, EKAR was developed thanks to an EGFP/mRFP FRET pair, a WWdomain and a substrate derived from Cdc25C, flanked by an ERK-specific docking domain [98].

Genetically-encoded FRET biosensors of several cell cycle regulating kinases have been described, and implemented to improve the overall understanding of their behavior. Noteworthy examples include the DNA-damage-sensing checkpoint kinase ATM [95], mitotic kinase CDK1/Cyclin B at the G2/M transition [97], Aurora B kinase during anaphase [90] and to probe Plk1 kinase activity upon checkpoint recovery [115].

More recently, FRET biosensors to probe kinase activities have been engineered with different AFP couples such as Venus, mTurquoise, Clover and mRuby, For example, Su, et.al developed a Src sensor based on mVenus/mKO FRET pair [125]. Moreover, since many kinases often exhibit cross-talk within a given signaling pathway and perform their functions in a concerted fashion or in interactive feedback loops, in response to activation of another kinase or enzymatic regulator, there is a growing interest to monitor several kinase activities simultaneously. Such multiparametric imaging strategies rely on the combination of fluorescent biosensors with FRET couples whose distinct spectral properties enable simultaneous imaging with different laser lines and spectral separation of signals following acquisition of multispectral data sets such as CFP/YFP and mOrange/mCherry couples, or CFP/YFP and mAmetrine/tdTomato [126,127,128,129].

Multispectral imaging modalities are becoming increasingly used in preclinical studies in animal models, to image disease and therapeutic response *in vivo*.

The development of imaging systems that allow the acquisition of multispectral data sets has allowed devising assays in which the behavior of several targets can be monitored by combining the simultaneous use of several biosensors [21,22,23]. Although multiparametric imaging remains challenging, it is particularly informative in drug discovery programs, as it allows gaining information on the specificity of pharmacological inhibitors, to characterize differences in potency and inhibitory kinetics, and to identify off-target effects. Multispectral imaging modalities are also becoming increasingly used in preclinical studies in animal models, to image disease and therapeutic response *in vivo*. Moreover, new generations of molecular imaging probes that combine several imaging modalities based on the electromagnetic properties of different wavelengths, multi-functional or multiplexed characteristics are being developed, thereby offering promising perspectives for diagnostics and drug discovery programs.

**Figure 8:**
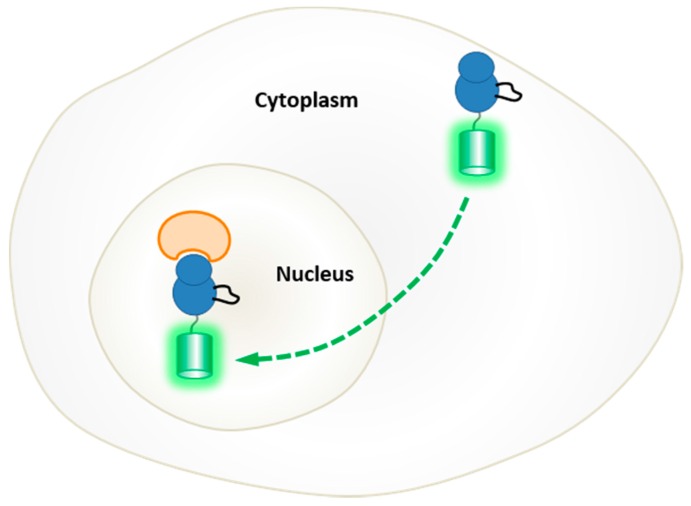
Positional biosensor of PKA. A different class of so-called positional biosensors was also developed to probe PKA activity and inhibition as a tool for high content screening [130]. This biosensor was designed by fusing GFP (Green Fluorescent Protein) to a domain that is recognized by the catalytic subunit of PKA, once it dissociates from its inhibitory regulatory subunit, which is further flanked by an NES (nuclear exclusion sequence) and an NLS (nuclear localization sequence). Upon binding, the catalytic subunit of PKA masks the NLS, thereby promoting nuclear export of the biosensor.

A different class of so-called positional biosensors was also developed to probe PKA activity and inhibition as a tool for high content screening [130]. This biosensor was designed by fusing GFP to a domain that is recognized by the catalytic subunit of PKA, once it dissociates from its inhibitory regulatory subunit, which is further flanked by an NES and an NLS. Upon binding, the catalytic subunit of PKA masks the NLS, thereby promoting nuclear export of the biosensor (Figure 8).

Although FRET-biosensors of PKs constitute powerful tools they are subject to a certain number of limitations. Ectopic expression requires time and may lead to significant differences in expression levels in a cellular population, unless cells are stably transfected. Moreover, many AFPs exhibit/suffer from strong pH sensitivities close to the physiological pH range, which may have dramatic consequences on fluorescence signals measured in living cells [87]. They may further be subject to oxidation, misfolding, aggregation or oligomerization, thereby failing to fluorescence, leading to decreased, unreliable or misleading interpretations [131]. In an attempt to circumvent these issues, efforts have been made to generate bright and inert variants of fluorescent proteins optimized for cellular applications in specific subcellular compartments [131].

## 5. Probing the Spatial and Temporal Dynamics of Protein Kinases in Living Cells

### 5.1. Genetically-Encoded Reporters of Protein Kinases

Protein Kinases exhibit spatial dynamics and frequently shuttle between different cellular compartments, which are characterized by different pH, viscosity and where different partners and substrates may be present and available for interaction and regulation (Figure 1c).

Several strategies enable to follow the spatial dynamics of PKs in living cells. The simplest form of imaging the spatio-temporal dynamics of protein kinases consists in engineering fusions of GFP or GFP-like genetically-encoded autofluorescent proteins (AFP) with the PK of interest. This strategy allows visualizing any gene product that can be genetically-encoded and expressed in fusion with an AFP [27,28,79,80,85,132,133,134,135,136]. Such fluorescent reporters or fluorescently-tagged PKs enable direct and straightforward imaging of dynamic changes in its subcellular localization following expression in mammalian cells.

Over the last decade, together with the development of a wide variety of AFPs, a wide variety of AFP fusions and imaging technologies have been developed to study gene expression, subcellular localization and dynamics [85], such as fluorescence recovery after photobleaching (FRAP), fluorescence loss in photobleaching (FLIP) to monitor the dynamics of proteins of interest within cells, FRET to monitor intracellular interactions between two ectopically expressed AFP fusions, fluorescence lifetime imaging (FLIM) to monitor changes in the local environment of a GFP fusion, for instance when it interacts with a partner, and fluorescence correlation spectroscopy (FCS) to gain information on the kinetics and thermodynamics of processes, typically changes in protein dynamics.

**Figure 9:**
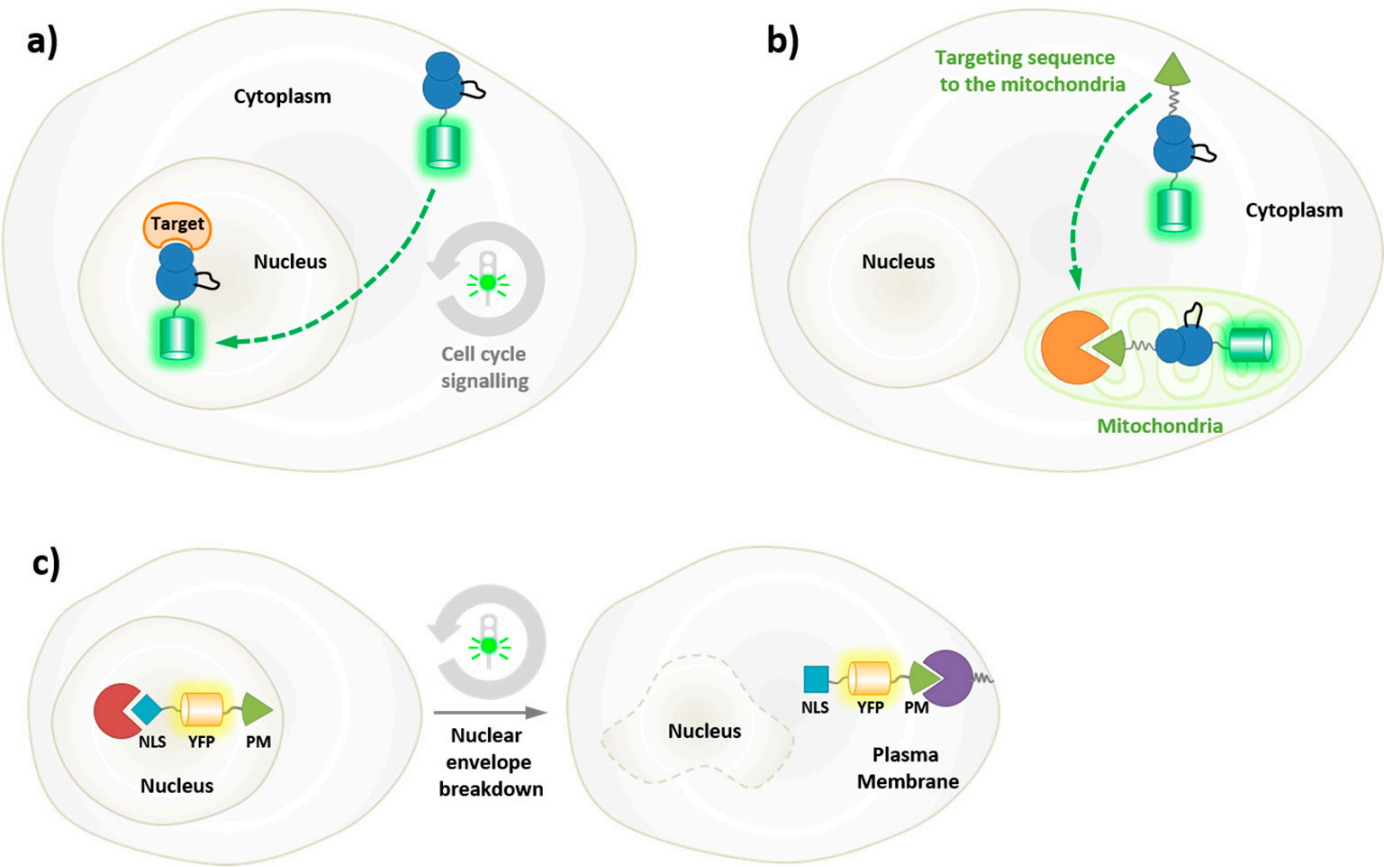
Fluorescent reporters of spatio-temporal kinase dynamics. Protein kinases shuttle in space and in time in a dynamic fashion in their cellular environment and between different subcellular compartments, (**a**) natural shuttling defined by spatio-temporal cell cycle signals; (**b**) organelle-targeting; and (**c**) cell cycle induced relocalization to PM.

In particular this approach has been widely implemented to study cell cycle regulated proteins [137]; for instance, to characterize the spatiotemporal shuttling of cyclins and cyclin-dependent kinases between the cytoplasm and the nucleus [138,139] (Figure 9a). Likewise, genetic fusions of AFPs with RTKs have enabled studies of their dynamics in the cell membrane [10]. Genetic reporters have further been implemented to image specific subcellular compartments by incorporating an organelle-targeting sequence (NES, NLS or mitochondrial localization sequence for example), which directs the expressed fusion to the desired intracellular location [140,141] (Figure 9b).

Cell-cycle markers, reporters of specific cell-cycle phases have been developed thanks to genetically-encoded fusions of fluorescent proteins functional elements that confer characteristic cell-cycle features such as cell-phase-dependent expression, nuclear translocation or proteolytic degradation [137]. For instance, a genetic construct encoding a YFP fusion with a plasma-membrane-targeting domain (PM) and a nuclear localization sequence (NLS) was engineered and implemented to monitor the kinetics between nuclear envelope breakdown and reformation in mitosis, since it localizes to the nucleus throughout interphase and relocates to the plasma membrane upon nuclear envelope breakdown [142] (Figure 9c). Similarly, a fusion of GFP with a fragment of cyclin B bearing a functional destruction box and a cytoplasmic retention signal (CRS), under control of its own promoter (CCNB1), was engineered to make a cell cycle marker which is expressed in Sphase, translocates into the nucleus at the G2/M transition following phosphorylation-induced inactivation of the CRS, and is degraded at mitosis [143]. Furthermore the combination of different AFPs fused to different proteins of interest enables the combined expression of two or more proteins whose subcellular localization differs in space and in time. This strategy was successfully implemented to develop the FUCCI (fluorescent ubiquitination-based cell-cycle indicator) system for studying the dynamic transitions between cell cycle phases in living cells, through combined use of RFP-Cdt1, a substrate of SCFSkp2 that accumulates in G1, with GFP-geminin, a substrate of APCCdh1 that accumulates in S/G2/M and is degraded in G1 [144].

### 5.2. Biorthogonal Labeling and Intracellular Labeling Strategies

**Figure 10:**
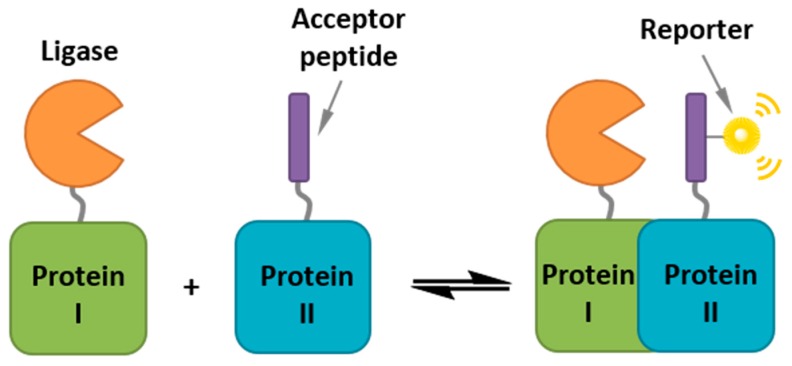
Intracellular Ligation.

Besides genetically-encoded fluorescent fusions of protein kinases, efforts have been made to develop strategies to label biomolecules directly with small molecule probes, thereby offering an alternative palette of fluorescent tools for cellular imaging [86]. Bioorthogonal labeling fluorescence labeling strategies involve chemical or enzymatic conjugation or crosslinking of fluorophores *in vitro* and *in vivo* (Figure 10) [145,146,147]. The first chemical tagging technologies reported involved incorporation of a peptide tag into the protein of interest, which could be specifically labeled with a synthetic fluorophore, such as the FlAsH peptide tag, the CLIP-, the TMP- or the SNAP-tag [148,149].

The challenge with bioorthogonal labeling strategies consists first in ensuring that the labeling reaction is sufficiently selective and second that the fluorophore is as suitable as possible to image intracellular proteins with high signal-to-noise ratios (bright, photostable, non-toxic and permeable across cellular membranes). Ideally the fluorescent probe should be non-fluorescent when unligated to the protein of interest, and unreacted probe should be easily washed out of cells. To this aim, more recent enzyme labeling strategies been developed to ensure complete and site-selective labeling, such as biotin ligase, transglutaminase, which labels a reactive glutamine within a specific recognition sequence, or a fluorophore ligase derived from *Escherichia coli* lipoic acid ligase (LplA), which has been engineered to catalyze rapid and specific intracellular ligation of synthetic 7-hydroxycoumarin, Pacific Blue, or resorufin to a 13mer transposable acceptor peptide within the protein of interest [150,151,152,153,154,155].

## 6. Probing the Conformational Dynamics of Protein Kinases

### 6.1. Conformational Dynamics of Protein Kinases

**Figure 11:**
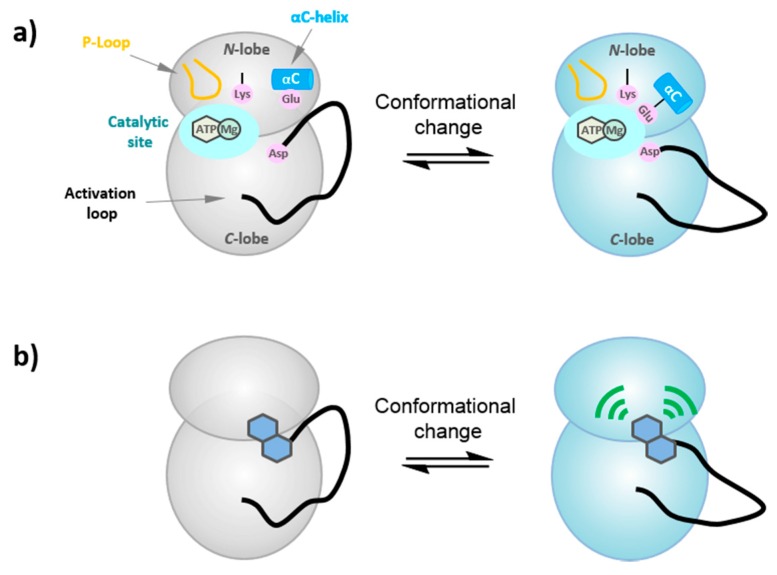
Conformation transitions in protein kinases. (**a**) In an inactive kinase the activation loop adopts a flexible, collapsed conformation, which undergoes a positional shift to assume a catalytically competent conformation in the active kinase, together with alpha helix C and the glycine-rich P-loop; the DFG-Asp motif is oriented into the active site where it coordinates ATP/Mg^2+^, the catalytic lysine forms a salt bridge with a glutamate in alphahelix C, and the positional shift of the T-loop enables substrate access and facilitates its binding to the catalytic cleft, where it is poised for phosphotransfer. (**b**) Fluorescent labels in kinases (“FLiK”) for the sensitive measurement of conformational changes in kinases upon ligand binding.

PKs lie at the heart of essential biological signaling pathways. Their activity is finely and tightly regulated at different levels and in distinct fashions, essentially through posttranslational modifications or protein/protein interactions [4]. Although these enzymes assume an overall highly conserved structure [2,3], they can adopt a variety of conformational states as a consequence of the different regulatory cues they are subject to [4,5,6,7,8,9]. Phosphorylation or partner binding alters the dynamics of interconversion between conformational states by inducing a concerted change in structural features. These conformational transitions have a direct consequence on protein kinase activity and function. Indeed, the position of several structural and regulatory elements, namely the alpha C helix, the glycine-rich phosphate binding P-loop within the N-terminal lobe of the PK and the activation loop (or T-loop), which comprises the DFG motif at its N-terminal tip, determine whether a kinase adopts an active or inactive conformation [4,6,8] (Figure 11a). The activation loop normally adopts a flexible, collapsed and dynamic conformation in the inactive kinase, but its phosphorylation stabilizes it in an ordered and extended conformation, locking it into an active conformation. Concomitantly, the alpha C helix, the P-loop and the activation loop assume a catalytically competent conformation, the DFG-Asp motif orientated into the active site where it coordinates ATP/Mg^2+^, while the catalytic lysine forms a salt bridge with a glutamate in alpha C helix, and the positional shift of the T-loop enables substrate access and facilitates its binding to the catalytic cleft, where it is poised for phosphotransfer. Protein kinase plasticity enables protein kinases to switch between inactive and active conformations in a dynamic fashion, in response to specific regulatory stimuli [5,6,9].

### 6.2. FLIK Technology

These dynamic features of PKs have been exploited to develop a conformation-sensitive assay known as FLIK for Fluorescence Labels in Kinases, based on incorporation of environmentally-sensitive fluorescent dyes at strategic positions close to or within dynamic segments in the kinase scaffold [156,157]. Such fluorescently-labeled kinases constitute sensitive probes that report on conformational changes between active and inactive forms of the kinase through changes in fluorescence. They constitute conformation-selective probes that for identification of modulators of protein kinase conformation and have been successfully implemented to screen for inhibitors that preferentially bind inactive conformations and prevent conversion of PKs into their catalytically active form. Indeed, FLIK technology was initially applied to screen for allosteric inhibitors of cSrc and MAPK that stabilize the inactive “DFG-out” conformation [158,159,160,161]. It was then further applied to identify inhibitors that bind the myristate pocket of Abl tyrosine kinase [162,163] (Figure 11b).Our group has developed a similar approach to engineer conformation-sensitive biosensors derived from CDK2 and CDK4, which respond to modulators of T-loop conformation, such as Cyclin A and D, respectively. Following incorporation of fluorescent labels into the C-terminal lobe of the kinase scaffold, these biosensors were successfully implemented to screen for allosteric inhibitors that target the T-loop of these kinases (personal communication).

## 7. Recent Developments and Cutting-Edge Approaches—What Is in the Pipeline?

Most of the technologies developed so far to probe the dynamic features of protein kinases rely on fluorescent proteins or synthetic dyes that absorb and emit in the visible range. However, this significantly limits their application for live-cell imaging due to phototoxicity, and makes them essentially unsuitable for *in vivo* applications. To circumvent these issues, efforts have been made to generate fluorescent proteins and probes, which can be used in the near-infrared and infrared wavelengths. Moreover, strategies to improve signal-to-noise ratio and control the activation of these probes with light are actively being pursued by numerous laboratories, offering cutting-edge technologies for new areas of investigation.

### 7.1. Near Infrared and Infrared Probes

In order to obtain high signal-to-noise ratio and spatial resolution while imaging in living cells or animal models, it is imperative to use fluorophores with long-wave excitation and emission spectral properties. Over the recent years efforts have been made to generate novel generations of fluorescent probes with photophysical properties best suited for *in vivo* studies [31,83,85,164,165,166,167]. Infrared (IR) and near-infrared (NIR) fluorescent probes are of particular interest for cellular imaging and *in vivo* applications [83,168,169,170,171,172,173,174,175,176]. Indeed when compared to visible light, NIR light is advantageous for biological imaging due to minimal photodamage to biological samples, deep tissue penetration, little scattering, and low background autofluorescence of seric proteins and other biomolecules in the NIR range, so that high contrast can be obtained between the imaging probe and background tissue. Although various NIR light-excitable fluorescent probes have been reported, most of them present limitations for bioimaging. In many cases they suffer from photobleaching, or do not emit adequate fluorescence in aqueous media due to molecular stacking or poor water-solubility [177,178]. Until recently, the only widely used NIR fluorophores were cyanine dyes (Cy5–Cy7), which have been extensively employed in the design of NIR fluorescent biosensors [179,180]. Recently, a new class of NIR wavelength-excitable fluorescent dyes based on the silicon-rhodamine scaffold has been developed, which has demonstrated superior characteristics for *in vivo* fluorescence imaging and live-cell super-resolution microscopy [181,182,183,184,185]. This class of probes demonstrate the potential of fluorescent scaffolds that are excitable in the NIR region, photostable, and soluble in aqueous media. 

Moreover, increasing attention has been paid to nanotechnology-based imaging probes. Quantum dots (QDs), nanocrystalline fluorophores with broad excitation spectrum and far-red to NIR emission, have been employed to develop a number a biological and *in vivo* imaging applications [186]. In addition to organic fluorophores and QDs, a variety of new nanomaterials are receiving attention for their NIR, luminescent or photoacoustic properties. The development of gold nanoparticles, semi-conductor quantum dots, polymer nanoparticles, carbon nanotubes, nanodiamonds, and graphene derivatives, with exceptional optical and spectroscopic properties, offer promising alternatives to both small-molecule and protein fluorophores for high-resolution imaging of intracellular processes and *in vivo* imaging [187,188].

### 7.2. Photoactivation Strategies

#### 7.2.1. Caged Compounds

**Figure 12:**
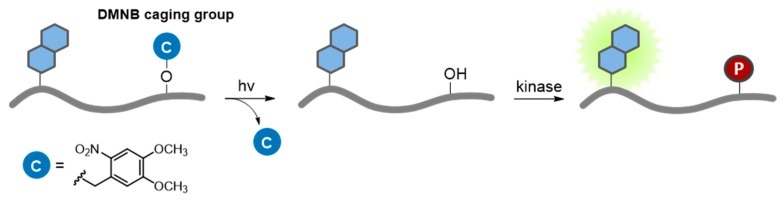
Photoactivatable biosensor. The residue to be phosphorylated by a kinase of interest is protected by a caged group (C), which can be removed by UV illumination, thereby enabling addition of a phosphate group (P).

The development of light-activated probes and biosensors has provided means of controlling the imaging probe, of “switching it on or off” through selective illumination. Photoactivation ensures that nonspecific noise attributable to basal fluorescence prior to activation is completely silenced, and a consequent enhancement of the specific signal corresponding to the target/event of interest. Light-activatable biosensors of protein kinases have been essentially engineered by molecular caging of the phosphorylatable residue (Ser, Thr or Tyr) with a photolabile group, which can be selectively released on a microsecond to millisecond time scale with long wavelength UV light (>350 nm) (Figure 12). This strategy offers the advantage of controlling both the timing and the relative concentration of biosensor released by selective photoactivation, after its penetration into living cells [189,190,191,192]. The most widely utilized caging groups used in biological studies are the *o*-nitrobenzyl systems and their derivatives, which have served for controlled release of a variety of small and macro-molecules [193,194]. For instance syntheses of photoprotected Ser and Tyr side chains with 4,5-dimethoxy-2-nitrobenzyl (DMNB) and their incorporation into kinase substrates have been described. A photoactivatable variant of the NBD-PKC probe (described in Section 3.2., reference 37) was engineered by masking the phosphorylatable serine group with DMNB, and successfully used to monitor PKC activity in HeLa cells following microinjection and selective photoactivation [55]. Furthermore, a caged PKC biosensor microinjected into PtK2 cells was applied to monitor PKC activity throughout mitosis with high spatial and temporal resolution, demonstrating the potential of light-controlled selective probe activation for imaging dynamic biological processes with high precision [56]. Similarly, a caged biosensor of Src allowed following the timing of kinase activity following microinjection and photoactivation in A549 cells [41]. Along the same lines, the quenching-based NIR PKA biosensor (described above) was used to monitor endogenous cAMP-dependent protein kinase activity in erythrocytes following light activation [45].

The most popular caging groups are photoreleased by long UV/short visible wavelengths (350–450 nm), which imposes serious restrictions to their application, since penetration into tissues is limited at these wavelengths and UV light can inflict cellular damage or death. In addition, the narrow window of the electromagnetic spectrum available for photoactivation limits the ability to control multiple cues by simultaneous orthogonal photoactivation of different caged compounds. Some of these concerns have been ameliorated through the use of two-photon excitation, which involves pulsed laser illumination with limited release in a small focal volume. Moreover, the Lawrence group has recently reported a tunable photo-responsive species based on the vitamin B12. The design of these compounds is based on conjugation of commercially available fluorophores to the cobalamin ring, which serve as antennas to capture long wavelength light, and promote scission of the Co–C bond with long visible and near-IR light (up to 800 nm) [195]. In this system, the corrin ring quenches the fluorescence of the conjugated fluorophores, thereby yielding stable and completely dark fluorophore-cobalamin conjugates until selective photolysis is triggered. This approach opens the door to the development of customized and sophisticated caged-based biosensors for the study of kinase dynamics *in vivo*.

#### 7.2.2. Photocontrollable Fluorescent Proteins—Photoswitching and Optogenetics

The development of fluorescent proteins that can be controlled by light has also provided improved means of imaging protein kinases with greater resolution, as well as new opportunities to investigate protein kinase function and regulation through precise control of their behavior in space and in time [196]. The development of genetically-encoded fluorescent protein species which are photoactivatable, subject to photoconversion between different states, reversibly photoswitchable and/or fast blinking, has been largely exploited to push the limits of spatial resolution thanks to the concomitant explosion of superresolution fluorescence imaging technologies, such as photoactivation-location and stochastic optical reconstruction microscopy [86,172,197].

The more recent development of optogenetic approaches enables manipulation of enzyme expression, activation or localization thanks to light. Genetically-encoded photoactuators and light-controlled optogenetic technologies first arose from studies in the neurobiology field which harnessed the power of light to control of cellular activity with precise spatial and temporal resolution [197,198,199,200,201,202,203]. These strategies rely on the use of naturally-occurring photoreceptors, light-sensing ion channels and ion pumps, as well as semi-synthetic chromophore-tethered receptors and photochromic synthetic ligands that can be used to induce and perturb biological processes in a controlled fashion [199,204,205,206].

**Figure 13:**
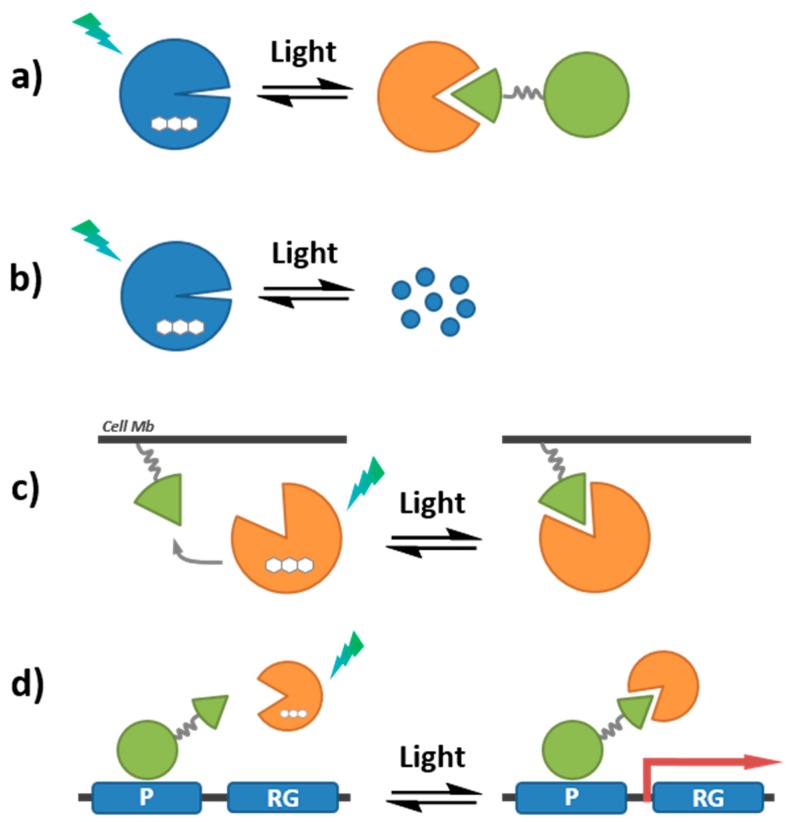
Optogenetic approaches to control protein function: (**a**) change in the activity of the protein; (**b**) protein degradation; (**c**) protein relocation; and (**d**) protein synthesis/gene expression (P = promoter; RG = reported gene).

A small number of “Opto-RTKs” have been engineered by fusing RTKs with light-activatable protein domains that undergo homodimerization in response to light. These optogenetic constructs constitute powerful tools to manipulate cell behavior, since they afford immediate and selective control, fast, reversible, time-and dose-dependent activation of RTKs through spatio-temporally precise induction dimerization, followed by propagation of intracellular signal cascades, mimicking mitogenic and morphogenic behavior normally induced by growth factors [207,208] (Figure 13).

One of the first optogenetic RTKs developed for mammalian cells was recently engineered by fusing the photoreceptor protein cryptochrome 2 (CRY2) to c-Raf, thereby allowing for blue-light reversible activation by heterodimerization of this kinase [207]. An alternative strategy was devised for selective activation of RTKs by low-intensity blue light-induced dimerization thanks to LOV (light-oxygen voltage)-sensing domains [208]. To this aim, the extracellular ligand-binding modules of FGFR1 (fibroblast Growth Factor Receptor), EGFR (epidermal growth factor receptor) and RET (rearranged during transfection) kinases were omitted to obtain chimeras that no longer respond to native signals, and LOV domains were fused to their intracellular catalytic domain.

### 7.3. Quenching-Based Activation Strategies

**Figure 14:**
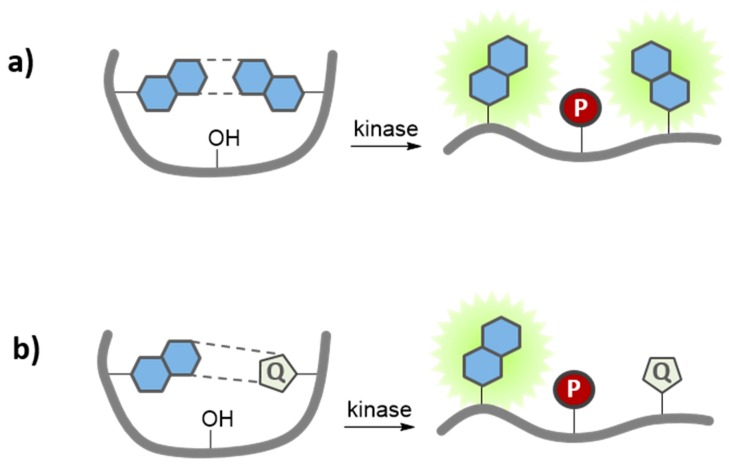
Quenching Strategies: (**a**) Quenching between fluorescent probes; and(**b**) Quenching of a fluorescent probe by a quencher group (Q). (P) phosphate group.

An alternative strategy to ensure a robust signal upon selective recognition of a target of interest consists in engineering reporters or biosensors which are silenced by quenching mechanisms, and selectively activated by the target (or its activity). These probes display minimal background and result in significantly higher signal-to-background ratios following activation by their target enzyme than conventional fluorescent probes [209,210,211]. Two strategies have been employed to quench probe fluorescence. The first involves intramolecular quenching between two fluorophores (through homo- or hetero-quenching) conjugated to the biosensor backbone (Figure 14a). The second involves quenching of a fluorescent probe by a synthetic quencher group, which is incorporated into the same scaffold (Figure 14b). Compared to homo-quenching activatable probes, this strategy can achieve higher activation ratios due to low preactivation fluorescence signal in the quenched state. However, this approach may exhibit limited fluorescence emission intensity relative to the former strategy in which there are a greater number of fluorophores. Furthermore, there are only a limited number of biocompatible quencher fluorophore pairs with sufficiently high activation ratios.

## 8. Concluding Remarks

Protein kinases are dynamic enzymes that play central roles in a wide variety of biological processes and that are notoriously subject to dysregulation, most often hyperactivation, in human disorders. The development of fluorescent probes, genetically-encoded reporters and fluorescent biosensors has provided means of studying the dynamic behavior of protein kinases in space and in time, of monitoring the kinetics of their activation and of probing conformational transitions associated with their activation.

The development of genetically-encoded fluorescent reporters of PKs has provided sensitive means of tracking these enzymes in their natural environment and to image spatio-temporal changes in their subcellular localization with high spatial and temporal resolution. The more recent intracellular labeling technologies enabling conjugation of small molecule fluorophores to targets within living cells, offer attractive alternatives to study endogenous PK targets with minimal perturbation. The design and engineering of fluorescent biosensors has afforded biologists and chemists with a toolbox of imaging probes to monitor oscillations of PK activities in living cells and organisms. Hence the opportunities offered by this panoply of tools and technologies are practically countless, but there are still major hurdles with respect to their application in living systems. Some of these limitations are associated with probe expression or delivery, probe brightness, stability, signal-to-noise ratio and the spectral window required for their application in a given context. However, with the more recent development of light-controlled fluorescent probes and strategies to control the activation of probes through quenching or molecular caging, yet another step has been made in the progress towards selective and high resolution imaging of dynamic kinase activities in biological signaling pathways. Furthermore, recent efforts to synthesize or engineer near-infrared and infrared probes and fluorescent probes clearly offer unmet expectations for *in vivo* imaging.

The ability to probe or image abnormal kinase activity, localization or conformation thanks to fluorescence-based technologies that monitor their dynamic behavior will undoubtedly open novel avenues for the development of personalized diagnostics. Fluorescent reporters and biosensors indeed offer attractive alternatives for detection of protein kinase biomarkers whose activity is dysregulated in pathological disorders or involved in disease pathogenesis. Furthermore these tools are perfectly suited for drug discovery programs and can be readily implemented in high throughput screening assays and postscreen characterization studies to gain insight into the mechanism of action and specificity of novel drugs.
